# Draft Genome Sequence of *Jiangella alkaliphila* KCTC 19222^T^, Isolated from Cave Soil in Jeju, Republic of Korea

**DOI:** 10.1128/genomeA.00721-15

**Published:** 2015-07-02

**Authors:** Jian-Yu Jiao, Lan Liu, Dong-Jin Park, Chang-Jin Kim, Min Xiao, Jing Chen, Li Li, Jing-Mei Zhong, Jiao Zhao, Wen-Jun Li

**Affiliations:** a Yunnan Institute of Microbiology, Yunnan University, Kunming, China; b State Key Laboratory of Biocontrol and Guangdong Key Laboratory of Plant Resources, School of Life Sciences, Sun Yat-sen University, Guangzhou, China; c The Affiliated Hospital of Kunming University of Science and Technology, Kunming, China; d Microbial Resource Center, KRIBB, Daejeon, Republic of Korea; e Beijing Genomics Institute at Shenzhen (BGI-Shenzhen), Shenzhen, China; f Key Laboratory of Biogeography and Bioresource in Arid Land, Xinjiang Institute of Ecology and Geography, Chinese Academy of Sciences, Urumqi, China

## Abstract

We report the draft genome sequence of *Jiangella alkaliphila* KCTC 19222^T^, isolated from cave soil in Jeju, Republic of Korea. This genome sequence, together with the previously sequenced *J. gansuensis* strain DSM 44835^T^, identified from a desert environmental source, will give us a better understanding of the school of “evolutionary taxonomy.”

## GENOME ANNOUNCEMENT

*Jiangella alkaliphila*, which belongs to the genus *Jiangella*, is an aerobic, Gram-positive, halotolerant filamentous actinomycete with a high G+C content (1). The genus *Jiangella* was first identified by Song et al. in 2005 (2), including four haloduric species currently on the List of Prokaryotic Names with Standing in Nomenclature (3). This taxon originally belonged to the family *Nocardioidaceae* within the suborder *Propionibacterineae* (2) and was finally placed in the family *Jiangellaceae* of the order *Jiangellales* under the class *Actinobacteria* (4). However, its position in the phylogenetic tree is still unstable in the actinobacterial areas using 16S rRNA information. We sequenced the genome of the type strain *Jiangella alkaliphila* KCTC 19222^T^ while considering that members of this group have been isolated from different environments (1, 2, 5, 6). This genome was compared with the previously sequenced *Jiangella gansuensis* DSM 44835^T^, which will provide further insight into the adaptation of these strains to different habitats and a better understanding of the school of “evolutionary taxonomy” (7).

Whole-genome sequencing of *Jiangella alkaliphila* strain KCTC 19222^T^ was completed using a paired-end sequencing method with the Illumina HiSeq 2000 platform (Illumina, San Diego, CA, USA); 1.76 Gb of data were produced, and 8,488,890 reads were used to assemble the daft genome by the SOAPdenovo2 package (8). The draft genome size of *J. alkaliphila* is 7,567,836 bp in 124 scaffolds, with an average length of 61,031 bp and an *N*_50_ scaffold length of 169,179 bp. Its G+C content is 72.18% according to the genome. Genes were predicted using Glimmer version 3.02 (9). The tRNAs and rRNAs were identified by tRNAscan-SE version 1.23 (10) and RNAmmer version 1.2 (11). About 7,238 protein-coding genes, 47 tRNAs, and 4 rRNAs were predicted from this assembly. The predicted coding sequences (CDSs) were translated and used to search against the KEGG and COG databases. These data sources were combined to assert a product description for each predicted protein.

The average nucleotide identity (ANI) was calculated between the genome sequences of *J. alkaliphila* KCTC 19222^T^ and *J. gansuensis* DSM 44835^T^ using the JSpecies software (http://www.imedea.uib.es/jspecies) (12) based on the BLAST method (ANIb) and the MUMer algorithm (ANIm). The result shows that the ANI values between *J. alkaliphila* and *J. gansuensis* were below 85.3%, which confirmed that *J. alkaliphila* was a new species belonging to the genus *Jiangella*, since ANI values of 95 to 96% correspond to the 70% DDH cutoff point recommended by Wayne et al. (13) for the delineation of bacterial species (14).

### Nucleotide sequence accession numbers.

This whole-genome shotgun project has been deposited in DDBJ/EMBL/GenBank under the accession number LBMC00000000. The version described in this paper is the first version, LBMC01000000.
